# MicroRNAs: Key Regulators to Understand Osteoclast Differentiation?

**DOI:** 10.3389/fimmu.2019.00375

**Published:** 2019-03-07

**Authors:** Claire Lozano, Isabelle Duroux-Richard, Hüseyin Firat, Eric Schordan, Florence Apparailly

**Affiliations:** ^1^IRMB, Univ Montpellier, INSERM, CHU Montpellier, Montpellier, France; ^2^Immunology Department, CHU Montpellier, Montpellier, France; ^3^Firalis SA, Molecular Diagnostics, Huningue, France

**Keywords:** microRNA, osteoclast, differentiation, regulation, detection, biomarker

## Abstract

MicroRNAs (miRNAs) are small non-coding single-stranded RNAs that represent important posttranscriptional regulators of protein-encoding genes. In particular, miRNAs play key roles in regulating cellular processes such as proliferation, migration, and cell differentiation. Recently, miRNAs emerged as critical regulators of osteoclasts (OCs) biology and have been involved in OCs pathogenic role in several disorders. OCs are multinucleated cells generated from myeloid precursors in the bone marrow, specialized in bone resorption. While there is a growing number of information on the cytokines and signaling pathways that are critical to control the differentiation of osteoclast precursors (OCPs) into mature OCs, the connection between OC differentiation steps and miRNAs is less well-understood. The present review will first summarize our current understanding of the miRNA-regulated pathways in the sequential steps required for OC formation, from the motility and migration of OCPs to the cell-cell fusion and the final formation of the actin ring and ruffled border in the functionally resorbing multinucleated OCs. Then, considering the difficulty of working on primary OCs and on the generation of robust data we will give an update on the most recent advances in the detection technologies for miRNAs quantification and how these are of particular interest for the understanding of OC biology and their use as potential biomarkers.

## Introduction

**Figure 1:**
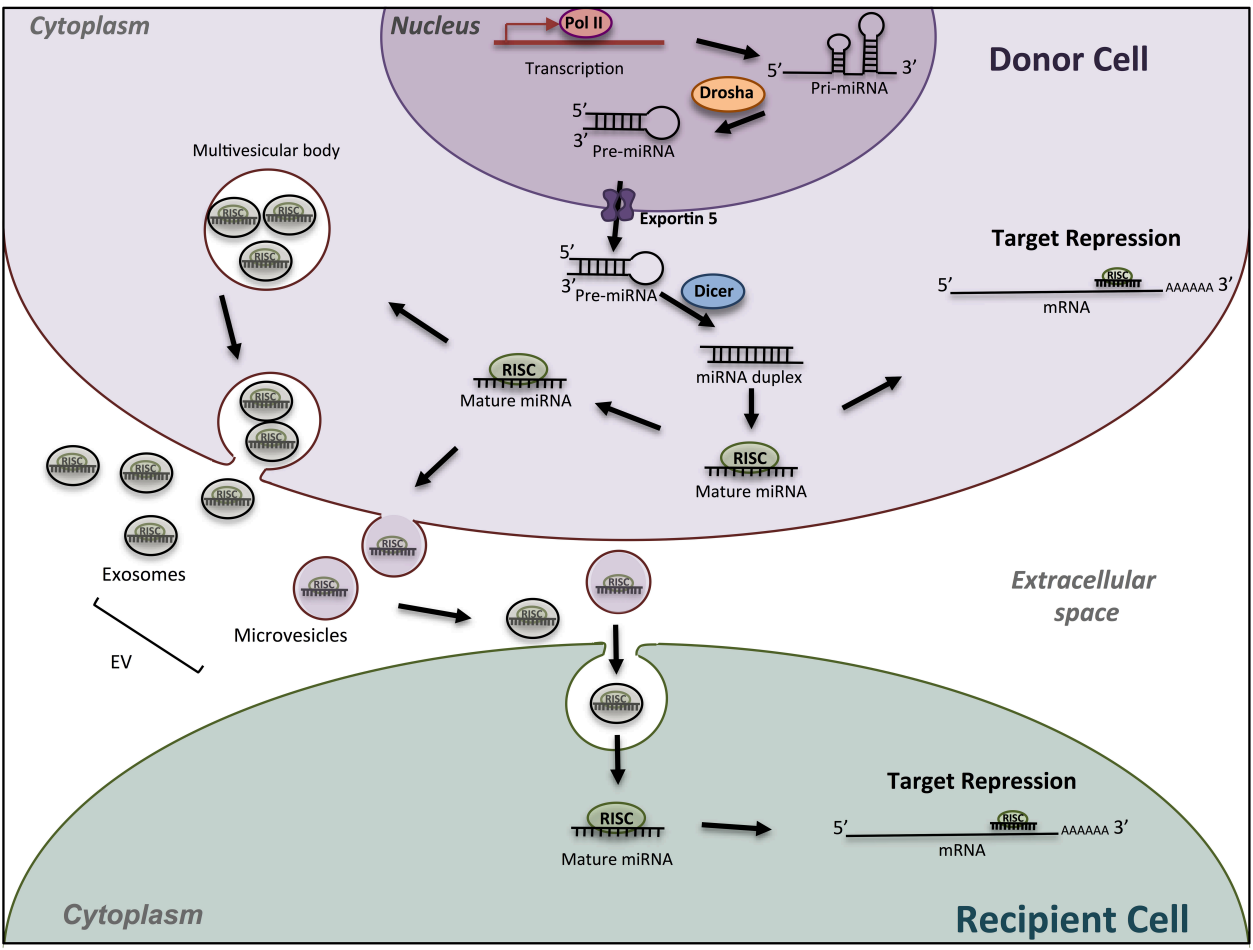
Schematic miRNA biogenesis and mode of action. miRNA biogenesis begins in the nucleus with transcription of miRNA gene into a pri-miRNA, followed by the action of the enzyme Drosha to produce pre-miRNA hairpins. After exportation into the cytosol, pre-miRNA, are processed into an intermediary miRNA duplex by Dicer. One miRNA strand is loaded onto the RNA-induced silencing complex (RISC) to form mature miRNA, which can regulate the expression of target mRNAs. The miRNA/RISC complex can also be incorporated into extracellular vesicles such as exosomes or microvesicle bodies, to be released into extracellular space. Then, miRNAs can be found in body fluids and travel across the entire body till elimination, or can be incorporated into a recipient cell and specifically regulate the expression of target genes into this new cell. Drosha, RNase III-type endonuclease family protein; Dicer, endoribonuclease; RISC; RNA- used silencing complex; EV, extracellular vesicles.

MicroRNAs (miRNAs) are key regulatory molecules that control cellular processes such as proliferation, migration and cell differentiation small. As shown in Figure 1, in the canonical pathway, miRNAs are transcribed by RNA polymerase II as large RNA precursors called primary (pri-) miRNAs that will be cleaved in the nucleus by the microprocessors complex into short hairpin precursors (pre-miRNAs) of about 70-nucleotides in length (1, 2). Pre-miRNAs are subsequently exported to the cytoplasm to be processed by DICER and yield mature miRNA duplexes (~22 nucleotides long) prior their loading onto the Argonaute-containing RNA-induced silencing complex (RISC). They bind through imperfect complementarity, mostly to the 3′-UTR regions of their target mRNAs, and lead to translation inhibition or degradation (3). These single-stranded RNAs thus modulate gene expression mostly at a posttranscriptional level. Current database describes more than 1,917 miRNA genes, which can contain 3 and 5p miRNAs. MiRNAs are recognized as crucial regulators of the expression of more than 60% of mammalian genes. The number of encoded miRNAs is limited compared to mRNAs and proteins expressed; however, one miRNA may regulate hundreds of mRNAs/lncRNAs and, as a result, may have substantial effects on gene expression networks. Although a lot of miRNAs have conserved sequences between species, the targeted mRNA sequences may be poorly conserved and biological effects are difficult to predict. *In silico* analyses using updated databases are thus useful to screen for putative targets according to the species and to find the miRNA sequence homology. *In vitro* functional studies are however needed to validate the miRNA targets, which may provide clues on the biological effects of the miRNA. In addition, available and freely accessible algorithms have been designed to identify potential miRNA-promoter interactions conserved between species that could represent additional clues to further push toward experimental validation (4). *In vivo* studies add indeed robustness to the biological role of miRNA-mediated regulation in pathophysiological conditions.

As to many other biological processes, miRNAs act as fine modulators to maintain bone homeostasis. Key evidence that miRNAs are essential to osteoclastogenesis is provided by genetic studies deleting one enzyme essential for their biogenesis, DICER1. DICER deficient mouse and osteoclast-specific DICER gene deficiency both lead to impaired osteoclast (OC) formation and activity (5, 6). Since then, the field of bone biology has regularly reviewed the role of miRNAs in OC biology or bone remodeling, mostly in the context of osteoporosis (7–13). A growing interest in miRNA-based therapeutic strategies has also emerged in bone-related disorders [for review see (14)]. Although the role of miRNAs in the OC lineage and ontogeny is a current hot topic, it remains poorly studied.

OCs are multinucleated cells specialized in bone resorption and derived from myeloid precursors that differentiate *in situ* in the bone marrow (15). The commitment of myeloid precursors in osteoclastic differentiation is controlled by micro-environmental factors to maintain bone homeostasis. Among them, osteoblasts, osteocytes and bone marrow stromal cells stimulate OC differentiation through the production of receptor activator of nuclear factor kappa-B ligand (RANKL), which binds to its receptor RANK. The growth factor macrophage colony-stimulating factor (MCSF) is also required to initiate the differentiation of osteoclastic precursors by binding its receptor CSF1R (colony stimulating factor 1 receptor). The Wnt-5a ligand is another pro-osteoclastic factor secreted by osteoblasts and OCs themselves (16, 17). In addition to these pro-osteoclastogenic factors, there are several inhibitory regulators including the osteoprotegerin (OPG), a soluble protein secreted by stromal cells that binds soluble and membrane forms of RANKL, thus preventing the activation of the RANK/RANKL signaling pathway. Indirectly, estrogens repress bone resorption by stimulating the production of OPG. Other environmental factors such as cytokines and lipid mediators impact on the osteoclastogenesis (18, 19). In addition to these well-known regulatory mechanisms, other elements could influence the commitment of myeloid precursors to the osteoclastic lineage. Instead of being differentiated into OCs, the myeloid precursors can also be differentiated into macrophages, especially in the presence of MCSF. Indeed, the osteoclastic and macrophagic lineages are thought to originate from an immediate bipotent precursor (20), demonstrating their close proximity. OCs retain the phagocytic potential and the ability to present the antigen of macrophages (21) but are the only cells capable of bone resorption. Among critical regulators of the polarization toward the OC vs. macrophage lineage, the mitochondrial metabolism has been involved (22). Although the role of miRNAs in the commitment of hematopoietic (23) and osteoblastic (24) lineages has been reviewed, only few studies have addressed the involvement of miRNAs in the commitment of the osteoclastic lineage.

The initiation of osteoclastogenesis requires the major signaling pathways RANK/RANKL and CSF1R/MCSF. The transcription factor NFATC1 (nuclear factor of activated T cells 1) is the cornerstone of the early phase of osteoclastogenesis. An amplification loop of NFATC1 induces the expression of many genes of the late phase, such as ACP5 (acid phosphatase 5, tartrate resistant), CTSK (Cathepsin K), and DCSTAMP (dendrocyte expressed seven transmembrane protein) [reviewed in (25)].

Under physiological conditions, the selective expression of miRNAs promoting and repressing the generation of OCs relies on the regulation of their own promoter (a shared promoter in case of miRNA clusters) and is thus closely linked to the sequential signaling pathways involved in the different steps of OC differentiation. MiRNAs are thus essential in a lot of biological feedback loops, including in the regulation of the OC biology.

Our present review will focus on the miRNA-mediated regulation of the sequential steps of OC formation. We will also report on the most recent advances in technologies used for the quantification of miRNAs. Finally, we will discuss the contribution of these technologies to the field of OC biology and the questions that remain to be asked.

## miRNAs and the Commitment of Progenitors Toward OCs

There are very few studies available on the miRNA-mediated regulation of OC commitment. Osteoclastogenesis is repressed by miR-155-mediated control of the transcription factor MITF (melanocyte inducing transcription factor) that is involved in the differentiation of monocytes toward macrophages (26). Conversely, miR-29 family (miR-29a, b, c) guides the commitment of bone marrow precursors toward the OC lineage by inhibiting GPR85 and CD93, two molecules involved in macrophage engagement (27). Authors showed that the transfection of the mouse monocytic cell line RAW264.7 with an inhibitor of miR-29 promotes macrophage differentiation, as evidenced by an up-regulation of the F4/80 surface marker and of the phagocytosis, even in presence of the major pro-osteoclastogenic cytokine RANKL.

## miRNAs in the Early Phase of OCs Generation

**Figure 2:**
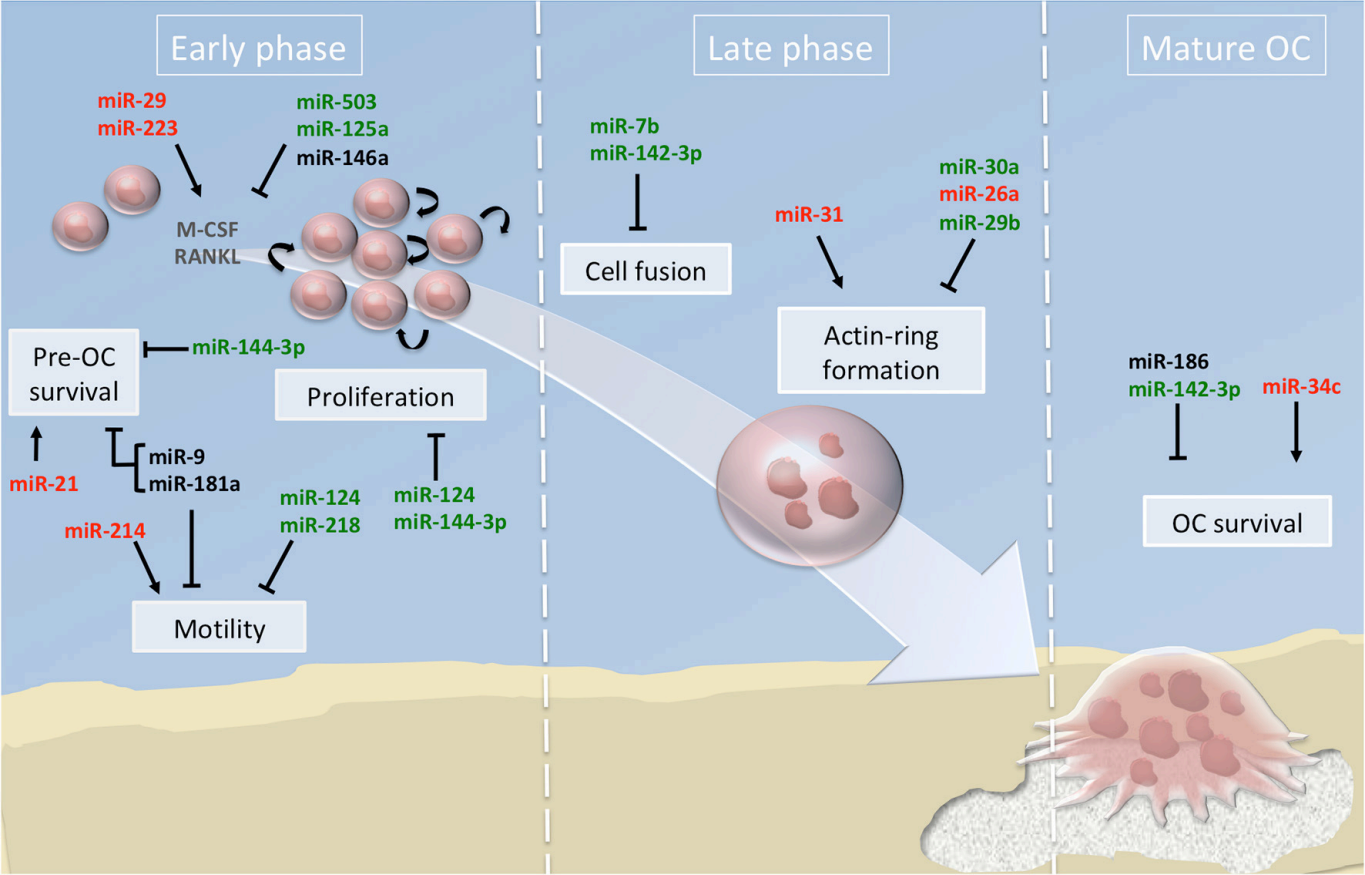
miRNA regulation of osteoclast differentiation. Illustration of the 3 phases of osteoclastogenesis. The early phase is associated with pre-osteoclast (OC) survival, motility, and proliferation; the late phase focused on pre-OC cell fusion and OC acting-ring formation; and the mature OC phase consists in the degradation of the mineralized matrix by mature OC. Green, red and black colors indicate down, up and normal miRNA expression, respectively. Arrows and bars indicate positive or negative effects on osteoclastogenesis respectively. M-CSF, macrophage colony-stimulating factor; RANKL, Receptor activator of NF-κB ligand.

Majority of the studies have described the global impact of miRNAs on the terminal OC formation, as evidenced by OC number and bone resorption activity. Few studies addressed beyond this final step which cellular processes are impacted. In Figure 2, we have summarized the miRNAs involved in the functional steps paving the generation of OCs, including cell survival, proliferation and motility of OC precursors. We also provide an updated list of their validated target genes (Table 1).

**Table 1:** MiRNAs involved in the early phase of OC generation.

**miRNAs**	**Species**	**Up/Down**	**Overall impact**	**Targets**	**Early steps impacted**	**References**
miR-21	Mouse^a, b^	Up	Positive	PDCD4 FASLG	Survival	(34–36)
miR-29 family	Mouse^a^	Up	Positive	NFIA CDC42 SRGAP2	ND	(27)
miR-148a	Human^a^ Mouse^a, b^	Up	Positive	MAFB	ND	(31)
miR-183	Mouse^a^	Up	Positive	HMOX1	ND	(38)
miR-199a-5p	Mouse^a^	Up	Positive	MAFB	ND	(30)
miR-214	Mouse^a, b^	Up	Positive	PTEN	Motility	(41)
miR-223	Mouse^a, b^	Up	Pos/neg	NFIA	ND	(5)
miR-9718	Mouse^a, b^	Up	Positive	PIAS3	ND	(32)
miR-34a	Mouse^a, b^ Human^a^	Down	Negative	TGIF2	NS (survival, proliferation)	(54)
miR-124	Mouse^a^ Rat^a, b^	Down	Negative	NFATC1	Proliferation, motility	(67, 68)
miR-125a	Human^a^	Down	Negative	TRAF6	ND	(48)
miR-141	Monkey^a, b^	Down	Negative	EPHA2 CALCR	ND	(56)
miR-144-3p	Human^a^	Down	Negative	RANK	Survival, proliferation	(47)
miR-145	Mouse^a, b^	Down	Negative	SMAD3	ND	(58)
miR-155	Mouse^a^	Down	Negative	SOCS1 MITF	ND	(61)
miR-155	Mouse^a^	Down	Positive	TAB2	ND	(63)
miR-218	Mouse^a^	Down	Negative	ND	Motility	(52)
miR-218	Mouse^a^	Down	Negative	TNFRSF1A	ND	(53)
miR-340	Mouse^a^	Down	Negative	MITF	ND	(65)
miR-503	Human^a^ Mouse^a, b^	Down	Negative	RANK	ND	(46)
miR-9; miR-181a	Mouse^a^	ND	Negative	CBL	Survival, motility	(45)
miR-146a	Human^a^ Mouse^a, b^	ND	Negative	TRAF6	ND	(50, 51)

*Species and experimental context of the model used are indicated (a, in vitro). Up- or down-regulation of respective miRNAs during OC generation and the overall impact on OC differentiation are given. We detailed steps impacted in OC precursors (pre-OC) such as cell survival, proliferation and motility. Validated targets are also listed. PDCD4, programmed cell death protein 4; FASLG, Fas ligand; NFIA, nuclear factor I A; CDC42, cell division cycle 42; SRGAP2, SLIT-ROBO Rho GTPase activating protein 2; MAFB, MAF bZIP transcription factor B; HMOX1, heme oxygenase 1; PTEN, phosphatase and tensin homolog; PIAS3, protein inhibitor of activated STAT3; TGIF2, TGFB induced factor homeobox 2; NFATC1, nuclear factor of activated T cells 1; TRAF6, TNF receptor associated factor 6; EphA2: EPH receptor A2; CALCR, calcitonin receptor; RANK, receptor activator of nuclear factor kappa-B; SMAD3, SMAD family member 3; SOCS1, suppressor of cytokine signaling 1; MITF, melanocyte inducing transcription factor; TAB2, TGF-beta activated kinase 1 binding protein 2; TNFRSF1A, TNF receptor superfamily member 1A; CBL, Cbl proto-oncogene. ND, not determined; NS, not significant*.

### Pro-Osteoclastogenic miRNAs

In addition to its role in the OC commitment of precursors, miR-29 family may promote migration of precursors since miR-29 neutralization inhibits the migration RAW264.7 cells (27). Furthermore, miR-29 family is involved in early phase of osteoclastogenesis by targeting NFIA (nuclear factor I A), a negative regulator of CSF1R (27). CSF1R is also indirectly induced by miR-223 through NFIA targeting as a positive feedback loop enhanced by the transcription factor PU.1 induced downstream of the MCSF/CSF1R (5). Same authors however previously reported contradictory findings using the same RAW264.7 cell line, as overexpression of miRNA-223 suppressed TRAP-positive OC formation (28). These later data were in agreement with a work performed on human peripheral blood mononuclear cells (PBMCs) (29). Bone loss is enhanced in arthritic condition due to activation of OC differentiation, and miR-223 is intensely expressed in rheumatoid arthritis (RA) synovium, particularly in monocyte/macrophage and CD4^+^ T-cell subsets (29). All these findings suggest an important role of miR-223 in the early phase of osteoclastogenesis, but an in-depth evaluation of the expression level of miR-223 under physiological osteoclastogenesis requires further studies.

The early phase of OC generation triggers increased NFATC1 expression, together with reduced expression of its three negative regulators MAFB (MAF bZIP transcription factor B), IRF8 (interferon regulatory factor 8), and BCL6 (BCL6, transcription repressor) (25). MAFB is a relevant target of miRNAs in OC precursors. Up-regulation of miR-199a-5p and miR-148a promotes the amplification loop of NFATC1 and the formation of resorbing OCs by targeting MAFB in RAW264.7 cells (30) and in CD14^+^ PBMCs (31), respectively. MiR-9718 is a newly described miRNA specifically expressed in the OC lineage, which promotes OC differentiation by targeting PIAS3 (protein inhibitor of activated STAT3) (32), another negative modulator of NFATC1 (33). Finally, the injection of molecules neutralizing miR-148a or miR-9718 in ovariectomy-induced osteoporotic mice increases total bone mass and decreases the OC number and activity (31, 32).

Among the other miRNAs up-regulated in OCs, the pro-osteoclastogenic role of miR-21 was demonstrated *in vivo* using the miR-21 KO mouse that display a slight increase in the trabecular bone mass and a reduced OC number and bone resorption (34). The expression of miR-21 is induced by c-Fos, which activation upon RANKL treatment of mouse bone marrow-derived macrophages (BMMs) operates a positive feedback loop by targeting the programmed cell death protein 4 (PDCD4) (35). The binding of c-Fos to miR-21 promoter is strikingly diminished by estrogen E2 treatment in RANKL-induced osteoclastogenesis. Estrogen attenuate miR-21 biogenesis, leading to increased FasL protein level and caspase-3 activity in mouse BMMs precursors (36). These results suggest that miR-21 expression is important in the development of OCs, particularly by controlling pre-osteoclast survival.

The activation of mitogen activated protein kinases (MAPKs) downstream of the early RANKL pro-osteoclastogenic signaling cascade is supported by the reactive oxygen species (ROS) produced by RANK-NADPH oxidase 1-dependant pathway (25). ROS production by mouse BMMs is regulated by heme oxygenase 1 (HMOX1), which attenuates osteoclastogenesis, specifically during the early phase of OC formation (37). MiR-183 is up-regulated by RANKL and targets HMOX1, thus promoting the early phase of osteoclastogenesis (38).

The PI3K/AKT pathway is induced by RANKL signaling and promotes cell survival (39). By reversing the action of PI3K (phosphoinositide 3-kinase), PTEN (phosphatase and tensin homolog) negatively impacts on OC precursor motility in the early phase of osteoclastogenesis (40). It was shown that miR-214 enhances the OC precursor differentiation via the PTEN/PI3K/AKT pathway, downstream of RANK signaling in RAW264.7 and primary mouse BMMs. *In vivo*, OC-specific miR-214 transgenic mice exhibit reduced expression of PTEN, increased OC resorption activity, and reduced bone mineral density (41). Since a miR-214/PTEN axis has been involved in cell proliferation and invasion of various cancer cells (42–44), it would be of particular interest to investigate the specific role of miR-214 on cell proliferation, survival and motility in the context of OC lineage.

The over-expression of miR-9 and miR-181a diminishes the migration of RAW264.7 cells and primary mouse OC survival by repressing the expression of the proto-oncogene Cbl, which enhances the amount of the pro-apoptotic protein Bim (45). This was the first study reporting a functional role for miR-9 and miR-181a by targeting proteins belonging to the apoptosis pathway. Further experiments are needed to confirm the potential role of these miRNAs on the precursor survival during osteoclastogenesis.

### miRNAs With an Inhibitory Role on OC Precursors

The initiation of the OC precursor differentiation is largely mediated by RANK/RANKL signaling. The expression density of RANK at the cell surface conditions the efficacy of RANK trimerization and downstream signal transduction. In human CD14^+^ precursors, miR-503 targets RANK mRNA in the coding sequence (CDS) region, leading to reduced RANK protein level, OC numbers and cell density *in vitro* (46). *In vivo*, treatment of OVX mice with anti-miR-503 reduced bone resorption (46). The 3′ untranslated region (UTR) of RANK is also targeted by miR-144-3p in CD14^+^ precursors, controlling OC formation, proliferation and survival of OC precursors (47).

The binding of RANKL to RANK induces the recruitment of the adaptor protein TRAF6 (TNF Receptor Associated Factor 6). This important signaling adaptor for RANK is targeted by miR-125a and miR-146a in human PBMCs (48, 49). The expression of miR-125a is controlled by NFATC1, which directly binds to the promoter of miR-125a during osteoclastogenesis and reduces its expression (48). MiR-146a has been extensively studied in monocytes in pathological conditions, including pathologies associated with bone erosion such as RA. The expression of miR-146a is induced by LPS, TNFα, or IL1β signaling cascades, through the activation of NF-κB (nuclear factor kappa B), which directly binds to the miR-146 promoter (49). It was shown that miR-146a inhibits OC formation from human PBMCs in a dose-dependent manner and that the treatment of collagen-induced arthritic mice with miR-146 mimics attenuates bone resorption (50). We recently demonstrated that the reduced expression of miR-146a in the Ly6C^high^ monocyte subset of arthritic mice is involved in the pathogenic bone erosion, and that it can be rescued by specific delivery of miR-146 mimics to Ly6C^high^ monocytes (51). Taken together, these findings evidence a negative regulation of osteoclastogenesis by NFκB-induced miR-146a to partly counterbalance the deregulated differentiation of OC precursors in inflammatory disorders.

The RANK/TRAF6 signaling cascade activates the NFκB and MAPK pathways, which may represent additional miRNA targets. Indeed, miR-218 over-expression inhibits osteoclastogenesis by controling the p38MAPK pathway in mouse BMMs (52). Interestingly, miR-218 negatively impacts on the migration of RANKL-treated BMMs. Although authors also reported a decrease of actin-ring formation, it was most probably a consequence than a cause of the decreased OC number. The putative targets of miR-218 were not explored in this study. A recent study confirmed the negative regulation of osteoclastogenesis by miR-218, and show that it was mediated by targeting TNFRSF1A (TNF receptor superfamily member 1A), which leads to the inhibition of the NFκB pathway activation in RAW264.7 cells (53). Overall, miR-218 may act on both NFκB and MAPK pathways in OC precursors to control OC precursor differentiation, and further studies are required to unravel the molecular mechanisms involved.

The early RANKL signaling is mediated by two transcription factors, NFκB and AP1, which are essential to the initiation of the NFATC1 amplification loop. AP1 components such as c-Jun and c-Fos could be critical targets to modulate NFATC1 activation in OC precursors. The transcriptional regulator TGIF2 (TGFB induced factor homeobox 2) is induced by NFATC1, c-Fos and c-Jun and potentiates the activity of NFATC1 and c-Jun in turn, promoting the osteoclastogenesis in a positive feedback loop (54). Interestingly, TGIF2 is a direct target of miR-34a, and OC-specific miR-34a transgenic mice exhibit lower bone resorption and higher bone mass, with no alteration of OC precursor survival and proliferation (54). Finally, miR-34a seems to negatively regulate the NFATC1 pathway during OC differentiation, mostly in the early phase upon RANKL signaling.

RANKL signaling enhances another co-stimulatory signal mediated by the ephrinA2-EphA2 interaction at the cell surface of OC precursors. EphrinA2 expression is rapidly induced in a c-Fos-dependent manner and cleaved by metalloproteinases to release an active soluble form able to interact with its receptor EphA2, enhancing osteoclastogenesis (55). In rhesus monkey BMMs EphA2 is a potential target of miR-141 (56). OC differentiation and bone resorption are suppressed *in vitro* by miR-141, and *in vivo* using repeated injections of an OC-targeted delivery system into aged monkeys (56). The calcitonin receptor (CALCR) is also a target of miR-141 in rhesus OCs. A down-regulation of CALCR is however expected to suppress the negative effect of calcitonin on OC differentiation and to enhance bone resorption, whereas miR-141 globally inhibits OC differentiation and activity, both *in vitro* and *in vivo*. One can speculate that the targeting of CALCR by miR-141 may represent a minor part of the effects of miR-141 functions in rhesus OCs.

Another critical interaction in RANKL-induced osteoclast ogenesis is the cooperation between Smad complex and c-Fos, which leads to NFATC1 transcription (57). Recently, miR-145 was shown to target Smad3, thus reducing the formation of p-Smad2/3 complex, repressing c-Fos and NFATC1 transcription in mouse OC precursors, and decreasing OC number (58). Smad proteins are induced by members of the transforming growth factor beta (TGFβ) super family, and Smad pathway has become of particular interest in inflammatory disorders [reviewed in (59)]. TGFβ1/Smad4 signaling directly induces miR-155 expression, a negative regulator of osteoclastogenesis (60). In addition, miR-155 is induced by interferon (IFN)-β and mediates its suppressive effect on OC differentiation by targeting the pro-osteoclastogenic gene SOCS1 (suppressor of cytokine signaling 1) in OC precursors (61). These data were suggestive of a suppressive role of miR-155 in osteoclastogenesis. In physiological condition, miR-155 is downregulated during osteoclastogenesis. Nevertheless, miR-155 is up-regulated in activated immune cells, such as lymphocyte B-cells, T-cells and dendritic cells, promoting the inflammatory response and thus aggravating the inflammatory-induced arthritis and bone erosion *in vivo* through the indirect immune-mediated activation of OCs [reviewed in (62)]. In lipopolysaccharide (LPS)-induced inflammatory condition, miR-155 directly induces autophagy in OCs as well as OC differentiation and activity by targeting TGFβ-activated kinase 1-binding protein 2 (TAB2) (63). A fine modulation of the TAB2 expression level may promote the destabilization of the inactive complex TAB2/Beclin1, leading to (i) the release of Beclin1 and induction of autophagy and (ii) the interaction of the adaptor proteins TAB2 and TAK1 that activates the RANK/TRAF6/NFκB pathway. These findings illustrate the variable role of miR-155 in osteoclastogenesis depending on the microenvironment, i.e., according to the presence of LPS, IFNβ, or TGFβ-mediated inflammatory signals. To make things even more complex, miR-155 also targets the transcription factor MITF that is up-regulated upon RANKL signaling (60, 61). MITF plays a critical role in the OC differentiation by collaborating with NFATC1 in the early phase of osteoclastogenesis (64). In summary, miR-155 seems to globally inhibit the OC generation in physiological condition by acting both in the commitment of myeloid precursors (26) and in osteoclastogenesis (61) through the regulation of MITF expression. MITF is also targeted by miR-340, which inhibits the OC differentiation of mouse BMMs (65). This reinforces the idea that MITF is a key target for miRNAs in osteoclastogenesis.

Finally, NFATC1 has hardly been described as a direct target of miRNAs so far. The activity of NFATC1 is regulated by phosphorylation, which retains NFATC1 in the cytosol compartment and inhibits its translocation into the nucleus. Epigenetic controls of the NFATC1 gene have been reported [reviewed in (66)]. To date, only miR-124 is predicted to bind NFATC1 in its 3′ UTR in OC precursors (67). The targeting of the 3′UTR of both rat and human NFATC1 mRNAs by miR-124 was confirmed using luciferase reporter assays (68). Functionally, miR-124 represses NFATC1 expression in mouse BMMs and diminishes the migration and proliferation of OC precursors (67), without impact on their survival (69).

## miRNAs in the Late Phase of OC Generation

To achieve OC maturation, the key transcription factors MITF, PU.1, and NFATC1 lead to the expression of several osteoclastogenic genes involved in the cytoskeleton organization, the cell fusion and actin-ring formation. MiRNAs also regulate the late stage of OC formation by targeting Rho GTPases, DCSTAMP, CSTK, and the RANKL-receptor inhibitor LGR4 (Table 2).

**Table 2:** MiRNAs involved in the late phase of OC generation.

**miRNAs**	**Species**	**Up/Down**	**Overall impact**	**Targets**	**Late steps impacted**	**References**
miR-26a	Mouse^a^	Up	Negative	CTGF	Actin ring formation	(82)
miR-31	Mouse^a^	Up	Positive	RHOA	Actin ring formation	(70)
miR-34c	Mouse^a^	Up	Positive	LGR4	OC survival	(74)
miR-7b	Mouse^a^	Down	Negative	DCSTAMP	Cell fusion	(79)
miR-29b	Human^a^	Down	Negative	FOS MMP2	Actin ring formation	(77)
miR-30a	Mouse^a^	Down	Negative	DCSTAMP	Actin ring formation	(80)
miR-124	Mouse^a^	Down	Negative	RAB27A	ND	(69)
miR-142-3p	Human^a^	Down	Negative	PRKCA	Cell fusion, OC survival	(83)
miR-186	Mouse^a^	ND	Negative	CTSK	OC survival	(84)

*Species of model used are indicated; whether experiments were performed in vitro (a) or in vivo (b) is specified with superscript letters. Up- or down-regulation of respective miRNAs during OC generation and the overall impact on OC differentiation are given. We detailed steps impacted in the late phase such as cell fusion, actin ring formation and the survival of mature OCs. Validated targets are also listed. CTGF, connective tissue growth factor; RHOA, ras homolog family member A; LGR4, leucine rich repeat containing G protein-coupled receptor 4; DCSTAMP, dendrocyte expressed seven transmembrane protein; FOS, Fos proto-oncogene, AP-1 transcription factor subunit; MMP2, matrix metallopeptidase 2; RAB27A, RAB27A, member RAS oncogene family; PRKCA, protein kinase C alpha; CTSK, cathepsin K. ND, not determined; NS, not significant*.

### Pro-Osteoclastogenic miRNAs

One of the most up-regulated miRNAs during osteoclastogenesis in mouse BMMs is miR-31. It specifically acts on the actin-ring formation (70). Neutralization of miR-31 impairs the matrix resorption and the ring-shaped OC formation, while cell-fusion is conserved. Increased RhoA activity and protein expression level are also observed in miR-31-deficient OC precursors, suggesting that RhoA is targeted by miR-31 in the OC lineage (70). Small GTPases of the Rho family (Rac1, Rac2, CDC42, RhoA, and RhoU) play important roles in the cell-fusion of OC precursors, podosome organization, migration, and polarization of mature OCs [reviewed in (71)]. RhoA controls the polymerization of actin, the turnover of podosomes, and the migration of OCs through the bone matrix (72). While a moderate level of RhoA activity is required to allow both stability of the sealing zone and bone resorption, RhoA over-activation or inhibition cause disassembly of the podosomes and thus impair the OC activity [reviewed in (73)]. Finally, miR-31 seems essential to OC maturation by finely modulating RhoA activity.

Another relevant miRNA in mature OC biology is miR-34c, which promotes the OC survival at the end of maturation by targeting the R-spondins receptor LGR4 (leucine rich repeat containing G protein-coupled receptor 4), also known as GPR48 (74). During the OC maturation, NFATC1 induces the expression of LGR4 at the cell surface to negatively regulate RANK/RANKL-signaling by a direct competition with RANK. The binding of RANKL to LGR4 activates the NFκB-inhibitor GSK3β, which results in OC apoptosis (75). These data suggest that miR-34c sustains the OC formation.

### miRNAs Displaying an Inhibitory Role in OC Maturation

Based on the study of Franceschetti et al. (27), we reviewed above the role of miR-29 family in the OC commitment and in the early phase of OC generation. The authors however also showed a late up-regulation of miR-29 (a, b, c) family members at the third day of RANKL-induced OC differentiation of mouse BMMs, suggesting another predominant role of miR-29 in the late phase of osteoclastogenesis. They found two targets involved in the cytoskeleton organization, CDC42 and SRGAP2 (SLIT-ROBO Rho GTPase Activating Protein 2), which present opposite effects. Indeed, SRGAP2 belongs to GAP family, which inactivates Rho GTPases such as CDC42 by increasing the intrinsic GTPase activity of Rho proteins (76). Rho GTPases are essential in the polarization and the podosome belt/sealing zone formation of functional OCs [reviewed in (17, 71)].The neutralization of miR-29 family has no effect on the formation of the actin-ring in mature RAW-derived OCs. A role for miR-29 family in the OC maturation remains thus questionable. Another study showed a down-regulation of miR-29b during OC generation, and demonstrated an inhibitory role of miR-29b in resorption and actin-ring formation in human CD14^+^-derived OCs (77). Considering only the multinucleated cells for their analysis, authors observed disarranged nodular actin spots in OCs over-expressing miR-29b, leading to a failure to form actin-rings. Although the authors did not perform a validation of the known targets of miR-29b in the OC lineage, they observed a significant down-regulation of c-Fos and MMP2 expression in miR-29b-transfected OCs at the end of the differentiation. The apparent discrepancies on the role of miR-29 family members and miR-29b could be partially explained by the chosen approach in the different studies, which rely either on gain-of function of miR-29b (77) or loss-of function of miR-29 family (27), as well as on the nature of OC precursors used (cell line vs. primary cells).

In addition to its negative effect on cell proliferation and motility in the early phase of osteoclatogenesis (67), miR-124 seems to act on the late phase of the differentiation by targeting Rab27a (69), a protein belonging to the small Rab GTPase family and involved in vesicle trafficking and resorbing activity of OCs (78). Notably, miR-124 is under-expressed in the OVX mouse model, which displays a deregulated resorbing activity (69).

DCSTAMP is a key protein involved in cell fusion. DCSTAMP is targeted by miR-7b and miR-30a in mouse BM precursors (79, 80). Mimics of miR-7b and miR-30a repress the expression level of DCSTAMP, decrease the OC number and inhibit matrix resorption. Conversely, anti-miR-7b promotes the OC formation and increase nuclei number in mature OC, suggesting an increase of cell fusion events (79). By measuring the membrane merge rate, the authors confirmed that overexpression of miR-7b in mouse BMMs significantly abrogates OC fusion (81). Anti-miR-30a enhances the actin-ring formation (80). Similarly, miR-26a attenuates the actin-ring formation and resorption in mouse BMMs. MiR-26a targets the connective tissue growth factor (CTGF), which induces and interacts with DCSTAMP (82). Contrary to miR-7b and miR-30a, miR-26a is upregulated in the late phase of osteoclastogenesis, suggesting a physiological regulation of multinucleation in the OC lineage. Thus, it will be of particular interest to evaluate the role of miR-30a and miR-26a on cell fusion.

During OC formation from human CD14^+^ progenitors, the enforced expression of miR-142-3p inhibits cell-to-cell contact, clustering and fusion events associated with the induction of OC apoptosis upon RANKL stimulation (83). The negative effect of miR-142-3p on OC fusion could be partially explained by the silencing of PKCα. Indeed, PKCα is involved in the microtubule and actin networks and is predicted as a putative target of miR-142-3p by prediction software. A decreased expression of anti-apoptotic factors downstream of PKCα might also explain the induction of cell death (83), however it has not been functionally explored yet.

Finally, miR-186 was newly described as a negative regulator of mature OC survival. Mimics of miR-186 induce caspase-3/7 activity and OC apoptosis in transfected RAW264.7-derived mature OCs (84). Moreover, miR-186 targets the CTSK gene (84) and probably represses the resorbing function of OCs, although it remains to be explored in functional assays.

## Advances in Technologies for the Quantification of miRNAs in Biological Tissues

MiRNAs are classically studied from the total mRNA (including small RNAs) extracted from the tissue of interest. As miRNAs are expressed in various cell types and tissues, it is necessary to well define the targeted sample and to control the purity of the elicited source of miRNAs. The study of miRNAs in primary OCs is challenging because of the localization of OCs into bone cavities, and thus a purified extract of primary OCs is very hard. Finally, primary OC precursors from blood sample or bone marrow are used to derive OCs in *in vivo* experiments. Nevertheless, the OC differentiation in culture is only partial without reaching a pure OC population. The obtained population includes all stages from the precursor to the mature OC. The study of miRNAs in such heterogeneous cell culture is not satisfactory and gives a lot of variations between samples that could impact on the reliability of the results, particularly in “end-point” studies without a kinetic expression of miRNAs during the OC differentiation. Some studies are based on purified OCs using chemical methods to eliminate non- and poor-adherent cells, as mature OCs are extremely adherent. Very recently, a novel method of purification based on the OC sorting has been described, allowing a standardized approach to better characterize the miRNA profiling in mature OCs (85).

Other biological tissues are become novel sources of study in the OC biology and the bone remodeling. Indeed, miRNAs can be exported from a donor cell to a recipient *via* exosomes and microvesicles (Figure 1), and thus participate to the cell communication between osteocytes, osteoblasts and osteoclasts in the bone micro-environment (86–89). Circulating miRNAs can also be used as biomarkers in pathophysiological conditions, reaching liquid biopsies as potential interesting samples in clinical application.

Molecular technologies are used to detect and quantify miRNAs, and some panels were developed to determine the miRNA profiling. Here we provide a global and detailed view of these technologies and their application in the quantification of miRNAs in biological tissues.

### Screening Methods for the miRNA Profiling

In recent years, technological advances in research tools including qPCR, microarrays, and next generation sequencing (NGS), have enabled sensitive detection of miRNAs. Typically, miRNA biomarkers are measured with quantitative polymerase chain reaction (qPCR) after RNA extraction and conversion into complementary DNA (cDNA). Many other methods are available for large screening of the miRNome to understand pathologies or discovery of biomarkers. Mestdagh et al., have extensively analyzed analytical parameters of many solutions available for miRNA measurement based on qPCR, hybridization platforms and sequencing technologies (90). Recently, new technologies for miRNAs measurement or optimization of existing methods have emerged. Currently, the miRbase 22 includes 1917 miRNA genes. With the constant evolution of the miRbase, the different miRNA detection platforms need to adapt their product. This flexibility however depends on the technic used. New technologies that are more sensitive, or extraction free chemistry, are definitively modifying the miRNA measurement landscape (Table 3).

**Table 3:** Technologies of miRNA profiling.

	**Exiqon qiagen LNA**	**Open array (Taqman)**	**Takara smarter microRNA-Seq kit**	**Illumina TruSeq**	**IonTorrent**	**Affymetrix miRNA 4.1**	**Agilent miRNA microarray (21.0)**	**Nanostring ncounter**	**HTG molecular**
Technology	qPCR	qPCR	NGS	NGS	NGS	Hybrid.	Hybrid.	Hybrid.	Hybrid. and NGS
Input RNA	40 ng	100 ng	100 ng	1000 ng	1000 ng	130 ng	100 ng	100 ng	15 μl/25 ng
Extraction	Yes	Yes	Yes	Yes	Yes	Yes	Yes	Yes	No
miRNAs in the assay	752	754	miRNome^*^	miRNome^*^	miRNome^*^	2,578	2,549	800	2,083

*LNA, locked nucleic acid; HTG, high throughput genomic; qPCR, quantitative polymerase chain reaction; NGS, next generation sequencing; Hybrid, hybridization*.

* *Based on current database miRBase 22 with 1,917 entries*.

Among these technologies, we will focus on the most innovative technologies for miRNA detection. These technologies provide real advance and alternative with the direct use of matrices avoiding preprocessing step or optimized library preparation for RNA-Seq method for the profiling of miRNAs. For the measurement of single miRNAs, new methods using microfluidic detection by laminar flow (91) or absolute quantification of signal by RCA-FRET technology (92) are in development. These last technologies may simplify the adoption of miRNA testing in clinical laboratories.

Screening methods such as the microarrays of Affymetrix (version 4.1) and Agilent (v21) now include 2,578 and 2,549 miRNAs, respectively, thus offering a more comprehensive dataset. In addition to these updated microarray versions, the company Takara Bio have recently developed the SMARTer miRNA-Seq kit, which uses Mono-Adapter ligation and Intramolecular Circularization (MAGIC) technology to efficiently capture miRNA species with reduced bias inherent to other approaches. Measurement of equimolar mixture of 963 miRNAs shows that >70% of the miRNAs are accurately represented, whereas other competitors have 49–79% of the miRNAs underrepresented Nevertheless, besides having less bias than the competitors (including TruSeq and NEXTFlex), this kit also produces large amounts of side products and as a result did not perform better for the detection of biological miRNAs (93). Since several years now, a new technology of HTG Molecular (High Throughput Genomic) called EdgeSeq provides miRNAs measurement without RNA extraction. The technology is a combination of hybridization with nuclease protection assay and next generation sequencing. Based on an extraction free chemistry, the technology^1^ allows the direct measurement of miRNAs from as little as 15 μl of body fluid. This last critical point avoids biases associated to the extraction protocols (94) and increases the sensitivity of the measure. Correlation between replicates is very similar to those shown previously (90) for hybridization and sequencing technologies, whereas accuracy of the gradient of miRNA measure is superior to other hybridization and sequencing platform^2^. Nevertheless, cross reactivity is higher compared to qPCR assays^2^. It has been shown that HTG EdgeSeq results were closest to the RNASeq results with >95% concordance on tissue samples (95). The technology also shows very good correlation with the PCR on plasma samples, with Pearson's coefficient of 0.93 and 0.94 for qPCR and digital PCR (dPCR) data, respectively (96).

### Circulating miRNAs as Biomarkers

In the past decade, the search for circulating miRNA for functional studies and biomarkers research has yielded numerous associations between miRNAs and different types of disease. However, many of these relations could not be replicated in subsequent studies under similar experimental conditions. Although this lack of reproducibility may be explained by variations in experimental design and analytical methods, guidelines of the most appropriate design and methods of analysis are scarce. MiRNAs have significant promise as biomarkers for diseases, due to their regulatory role in many cellular processes, and their stability in samples such as plasma and serum. Circulating miRNAs are moreover easily accessible. Biomarker experiments generally consist of a discovery phase and a validation phase. In the discovery phase, typically hundreds of miRNAs are simultaneously measured to identify candidates. Because of the costs of such high-throughput experiments, numbers of subjects are often too small, which can lead to false positives and negatives. In validation phase, a small number of identified candidates are measured in a large cohort, generally using quantitative PCR (qPCR). Although qPCR is a sensitive method to measure miRNAs in the circulation, experimental design, and qPCR data analysis remain challenging with many sources of biases. The MIQE guidelines are useful to stress on the most important biases in qPCR experiments and to give some elements to improve experimental practice (97). There is still a need for standardization or development of new methods to reach the clinic. Thus, choosing the right tools is critical for a successful miRNA-based experiment.

Despite new advances and evolution of technologies, many challenges remain unmet. For example, the impact of RNA isolation, which is known to induce biases (94), but also the lack of standardization of miRNA measurement or normalization of miRNAs data from plasma/serum samples, are key factors of variation, making more challenging the translation of biomarker discovery to diagnostic tool. Indeed, while many reports are describing miRNAs as potential biomarkers since many years, miRNA-based diagnostics have many difficulties to enter to the clinic and to get IVD approval. Moreover, these kinds of tests are likely best suited to a companion role. In contrast to RNA or DNA-based tests, especially that indicate the presence of SNP or a specific expression profile (98), miRNA tests produce results that are more difficult to interpret. While many miRNAs were reported as biomarkers in many reports (99), most miRNAs are expressed widely in a non-cell-specific manner, and their levels of expression are not differing drastically between patients' group and controls. For liquid biopsies such as blood, urine, or other body fluids, miRNA levels are very sensitive to pre-processing and post-processing factors. As a result, despite being very stable, miRNA-based tests are often based on a combination of miRNAs associated with an algorithm, and strict standardization of the entire process, from obtaining and processing the sample to results reporting, is mandatory and is key for reproducible results, no matter what technology is used.

Point of care diagnostics requires short detection time, small sample volume and portability of the device. These requests are often not compatible with miRNA measurement technologies since they are requesting many steps and devices. New methods based on microfluidic chip and laminar flow assisted dentritic amplification is currently under development, with a promise of time to result of only 20 min (91). Sensitivity and accuracy have still to be increased, but this attractive solution could potentially allow the compatibility of miRNAs to short diagnostic delay. Without such solution able to reduce time to results, use of miRNAs in diagnostic is only possible for non-urgent test. Most used platforms remain qPCR, with various technologies available such as LNA/Taqman/SybrGreen PCR, which request high level of standardization to provide accurate and stable results. Another novel technology that could facilitate miRNA transfer to clinic is called RCA-FRET, which is a combination of rolling circle amplification (RCA) and Förster resonance energy transfer (FRET) (92). Key advantage of the technology is a simple workflow, device needed and material for absolute quantification results. This last point is of key importance since normalization of miRNA data is a true hurdle.

Indeed, accurate quantification of miRNAs using qPCR is largely dependent on proper normalization techniques, the absence of which can lead to misinterpretation of data and incorrect conclusions (100). The goal of most miRNA experiments using qPCR is to identify differences in expression between two groups of samples. In this case, cell-free miRNAs from biofluids are emerging as important noninvasive biomarkers because of their stability. Many works are describing miRNAs as potential blood biomarkers. One challenge in studying miRNAs from serum and plasma is their relatively low abundance and lack of reliable endogenous controls. It has been shown that hsa-miR-24, hsamiR-126, hsa-miR-484 (101), hsa-miR-16-5p, hsa-miR-93-5p (102), hsamiR-484, and hsa-miR-191-5p (103) are stable normalizers in serum. Nevertheless, some reports indicate also these miRNAs not as normalizers but as biomarkers, as for miR-16-5p described in the progression of gastric cancer for example (104). In the absence of reliable endogenous controls in serum/plasma, exogenous or spike-in controls can be used to normalize miRNA expression data. Exogenous controls can also be used to monitor extraction efficiency or sample input amount for difficult samples (e.g., serum, plasma, or other biofluids).

In addition to most common used matrices, extracellular vesicles (EVs) are emerging and even more challenging to get reliable results. EVs are nanometer-scale particles, which include exosomes, microvesicles, and apoptotic bodies. EVs are intercellular communicators released by most cell types with key functions in physiological and pathological processes (86, 105). EVs deliver specific proteins, microRNAs and other cellular components. One of the most important aspects of EV research is analyzing their nucleic acid cargo, particularly miRNAs. These are commonly quantified by RT-qPCR or, increasingly, by comprehensive transcriptomic profiling using NGS or hybridization technologies. One critical issue is the methods for RNA extraction that can influence downstream analyses by yielding non-identical, kit-specific results. This is of particular challenge since typical concentration of this type of sample is low, RNA integrity is decreased and associated to high individual variability. Several kits are on the market and tested (106) but optimal isolation methodology is mainly dependent on the respective research setting and downstream analyses.

Overall, it is not easy to give a simple answer to a complex problem. Many methods are available for miRNAs measurement and generate complex data that need to be validated. Real efforts have to be done on standardization and analytical validation until one can consider to successfully translate biomarker discovery to the clinic.

## Conclusion

In the present review we have shown the complexity of understanding OC biology and pointed out key miRNAs that have been involved in OC differentiation as key regulators, with very specific roles in the different phases progressing from early progenitors toward fully mature OC. We also stressed the challenge to get reliable data from miRNA measurement either for supporting the knowledge of OC regulation or the translation into biomarker tools for clinical application.

## Data Availability

All datasets generated for this study are included in the manuscript and/or the supplementary files.

## Author Contributions

CL, ID-R, HF, ES, and FA wrote the review. CL, ID-R, and ES designed the figures and tables.

### Conflict of Interest Statement

The authors declare that the research was conducted in the absence of any commercial or financial relationships that could be construed as a potential conflict of interest.

## Footnotes

^1^From www.takarabio.com/learning-centers/next-generation-sequencing/technical-notes/accurate-mirna-representation-in-microrna-seq,2018

^2^From the poster presented at ESHG, 2017, available on https://www.htgmolecular.com/assets/htg/publications/PO-17-Aissaoui-EHSG-miRNA-Metrics.pdf
